# Adolescents’ Reactions to Adverts for Fast-Food and Confectionery Brands That are High in Fat, Salt, and/or Sugar (HFSS), and Possible Implications for Future Research and Regulation: Findings from a Cross-Sectional Survey of 11–19 Year Olds in the United Kingdom

**DOI:** 10.3390/ijerph17051689

**Published:** 2020-03-05

**Authors:** Nathan Critchlow, Jessica Newberry Le Vay, Anne Marie MacKintosh, Lucie Hooper, Christopher Thomas, Jyotsna Vohra

**Affiliations:** 1 Institute for Social Marketing and Health, Faculty of Health Sciences and Sport, University of Stirling, Stirling FK9 4LA, UK; a.m.mackintosh@stir.ac.uk; 2 Cancer Policy Research Centre, Cancer Research UK, 2 Redman Place, London E20 1JQ, UK; jessica.newberrylevay@cancer.org.uk (J.N.L.V.); lucie.hooper@cancer.org.uk (L.H.); chris.thomas@cancer.org.uk (C.T.); jyotsna.vohra@cancer.org.uk (J.V.)

**Keywords:** Marketing, advertising, HFSS, junk food, reactions, obesity, overweight, adolescents, food policy, regulation

## Abstract

The influence that marketing for foods high in fat, salt, and/or sugar (HFSS) has on adolescents extends beyond a dose-response relationship between exposure and consumption. It is also important to explore how marketing shapes or reinforces product/brand attitudes, and whether this varies by demography and Body Mass Index (BMI). To examine this, a cross-sectional survey was conducted with 11–19 year olds in the United Kingdom (*n* = 3348). Participants watched 30 s video adverts for a fast-food and confectionery brand. For each advert, participants reported reactions on eight measures (e.g., 1 = Makes [product] seem unpopular choice–5 = Makes [product] seem popular choice), which were binary coded based on whether a positive reaction was reported (*Yes/No*). At least half of adolescents had positive reactions to both adverts for 5/8 measures. Positive reactions had associations with age, gender and, to a lesser extent, BMI. For example, 11–15 year olds were more likely than 16–19 year olds to report appeal to their age group for the fast-food (*OR* = 1.33, 95% CI: 1.13–1.58) and confectionery advert (*OR =* 1.79, 95% CI: 1.51–2.11). If these reactions are typical of other HFSS products, future research and regulatory change should examine whether additional controls on the content of HFSS marketing, for example mandated health or nutritional information and revised definitions of youth appeal, offer additional protection to young people.

## 1. Introduction

Adolescents in the United Kingdom (UK) are exposed to a variety of marketing activities for food and drinks that are high in fat, salt, and/or sugar (hereafter ‘HFSS foods’), ranging from mass media advertising to subtle marketing activities (e.g., sponsorship and celebrity endorsement) [1,2]. Specifically, in a previous study, nine out of ten 11–19 year olds in the UK recalled seeing at least one marketing activity for HFSS foods in the past month and around one in six recalled seeing television adverts, social media adverts, or special price offers every day [1]. Reviews of research consistently suggest that exposure to marketing for HFSS foods has a consequential impact on dietary-related attitudes and behavior among children and adolescents, including increased consumption, encouraging parents to purchase (i.e., ‘pester power’), and poorer nutritional knowledge [1,3,4,5,6]. 

The influence that marketing for HFSS foods has on children and adolescents extends beyond a dose-response relationship between exposure and consumption. Instead, both theoretical and applied consumer research which has sought to rationalize how marketing influences consumption—such as studies based on the message interpretation process model—suggests that emotional and cognitive reactions to marketing, and how marketing shapes or reinforces product and/or brand attitudes, also play an important role in influencing behavior among young people [7,8,9,10]. A cross-sectional survey of 18–24 year olds in Australia, for example, found that the association between energy drink marketing and consumption was mediated through positive product attitudes (e.g., the belief they improve physical and mental performance) and subjective norms about consumption (e.g., the belief they are consumed by peers) [11]. Experimental research with both children and adolescents has also consistently shown that exposure to advertising for HFSS foods is associated with positive attitudes towards, and stronger attachments to, the brands depicted [5,12,13,14]. Moreover, qualitative research has also demonstrated that children and adolescents in the UK consider the marketing of HFSS foods to stimulate curiosity, hold important cultural and social capital, and normalize and socialize consumption [15,16,17,18,19,20]. 

Content analysis research, which focuses on the marketing output as the unit of analysis, highlights at least three ways in which the marketing of HFSS foods may elicit positive emotional responses or shape product and/or brand perceptions among children and adolescents. First, studies have shown that marketing for HFSS foods often uses attractive, fun, and engaging design features, such as visually stimulating graphics, branded characters, auditory cues, and immersive advergames [4,21,22,23,24]. Second, marketing also reportedly features content which may resonate with, or create a positive emotional response among, children and adolescents. This includes references to celebrities and popular culture, featuring brand equity characters (e.g., cartoon brand mascots), linking the brand to attractive and desirable identities or lifestyles, or featuring humorous content [21,22,23,24]. Third, marketing may place a greater emphasis on the sensory or emotive benefits of a product (e.g., taste or texture), as opposed to presenting objective information about nutrition and health, or may use ambiguous language that implies greater nutritional benefit than is actually present (e.g., fruit-flavored) [25,26,27]. 

In the UK, existing statutory controls prohibit advertising for HFSS foods on television during children’s programs or programs with a disproportionately high child audience [28]. In 2019, the UK Government also consulted on introducing additional placement restrictions, such as limiting television and online advertising for HFSS foods to 21:30–05:30 (i.e., a ‘watershed’); the outcome of this consultation is due to be announced in 2020 [1,29,30]. Where marketing activities for HFSS foods are permitted, content is self-regulated by a combination of the advertising and marketing industries and the food and drink producers [31]. Examples of stipulations in their current self-regulatory codes include ensuring the veracity of nutrition or health claims, not condoning or encouraging excessive consumption or poor nutritional habits, and not directly appealing to those under 16 years old through the selection of media or the context in which the marketing appears [32]. Critics, however, argue that the UK’s self-regulatory framework does not use objective criteria to determine youth appeal [33], which leads to inconsistent or subjective judgements [34]. It is also argued that the self-regulatory approach lacks expert knowledge and independent advice on what appeals to children and adolescents, and that the process of adjudication does not adequately take into account the relative expertise of young people and their parents about how marketing appeals to them and how it may shape product-, brand-, and nutrition-related attitudes [31,33]. 

Although existing research suggests that adolescents in the UK do have positive attitudes towards marketing for HFSS foods [15,16,17,18,19,20], much of this evidence is based on qualitative studies which lack the ability to generalize to larger samples, or content analyses which are only able to hypothesize (but not show) appeal. In this study, we therefore examine how adolescents react to two adverts for HFSS foods using a demographically representative sample of 11–19 year olds in the UK. By doing so, we emphasize the importance of involving their relative expertise when adjudicating to what extent, if at all, advertising appeals to them and how marketing may shape or reinforce their attitudes towards HFSS products/brands. We also examine to what extent, if at all, advert reactions are associated with demography and the Body Mass Index (BMI), to explore whether some adolescents may be more susceptible to the influence of the advertising of HFSS foods. We use these data to identify and discuss three avenues for future research and practice that may help to improve the efficacy of regulation and offer better protection to children and adolescents. 

## 2. Materials and Methods 

### 2.1. Design and Recruitment

The data were obtained from the 2017 Youth Obesity Policy Survey, an online cross-sectional survey with 11–19 year olds in the UK (*n* = 3348), conducted between April and May 2017 [1,35]. The survey was hosted by YouGov, a market research company, who recruited a sample intended to be representative of the UK population from their online panel. Participants aged under 16 years were approached through e-mails to existing adult panel members (e.g., their parents), while participants aged >16 years were approached directly via e-mails. Age (i.e., 11–19 years old) and membership of the online panel were the only inclusion criteria. Respondents received points on their YouGov account in return for participation (e.g., 50 points is equivalent to £0.50), which can be deemed for monetary value once a threshold is reached. A survey weight enabled descriptive data to be representative of the UK population (based on age, gender, ethnicity, region, and social grade).

### 2.2. Advertising Creatives for HFSS Foods

Participants were shown two adverts for HFSS food brands: (1) a fast-food brand and (2) a confectionery (sweets) brand that is an affordable with ‘pocket money’ brand (i.e., a small amount of money typically given to young people on a regular basis for minor expenses). Due to available space in the survey, which covered a variety of topics related to diet and health [1,35], we were limited to two HFSS adverts. We deliberately chose adverts that did not contain price offers, promotions, or competitions, which may have influenced reactions. These two adverts were chosen to reflect well-known brands whose products would typically be classified as HFSS by the UK Department for Health’s Nutrient Profiling Model (NPM) [36] and/or were included in Public Health England’s Sugar Reduction Programme [37]. Video adverts were chosen as they replicated content suitable for both television (e.g., advertising/commercial break) and online placement (e.g., posted on social media). These are two marketing activities through which adolescents in the UK report frequently seeing the marketing of HFSS foods [1] and are activities reportedly associated with an increased consumption of HFSS food and BMI among adolescents [1,38,39,40]. Neither advert had knowingly been subject to prior complaints, adjudications, or informal resolutions through the UK’s self-regulatory system [41]. Both adverts were from 2016—the year before data collection. 

**Figure 1:**
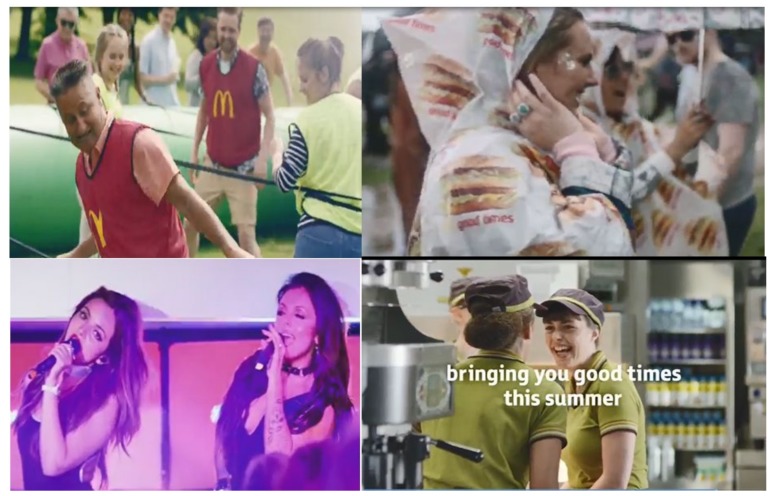
Stills from McDonald’s ‘*Summer of Good Times*’ advert (from https://www.youtube.com/watch?v=EfU-NV1H8FQ).

The first advert, from McDonald’s and lasting 30 s, represented a fast-food brand. The advert, entitled ‘The Summer of Good Times’ [42], depicted people engaged in a variety of activities traditionally associated with the British summertime, including going to the beach, playing football in a park, a summer fair (or fête), and a music concert (including footage of a performance by UK female pop group, ‘Little Mix’). The advert ends with consumers and staff depicted in a McDonald’s restaurant. The consumers are ordering and eating, while the staff appear to be socializing (Figure 1). Throughout, the advert features a diverse range of ages, ethnicities, and genders. With the exception of ‘Little Mix’, all other characters are actors. The final frame includes the slogan “Good times”, the McDonald’s logo, and social media hashtag “#hellogoodtimes”. An upbeat accompanying song (in an acoustic folk style) plays throughout, with lyrics congruent to the advert narrative (e.g., “Life is upon us, and the time for love is here and now”).

**Figure 2:**
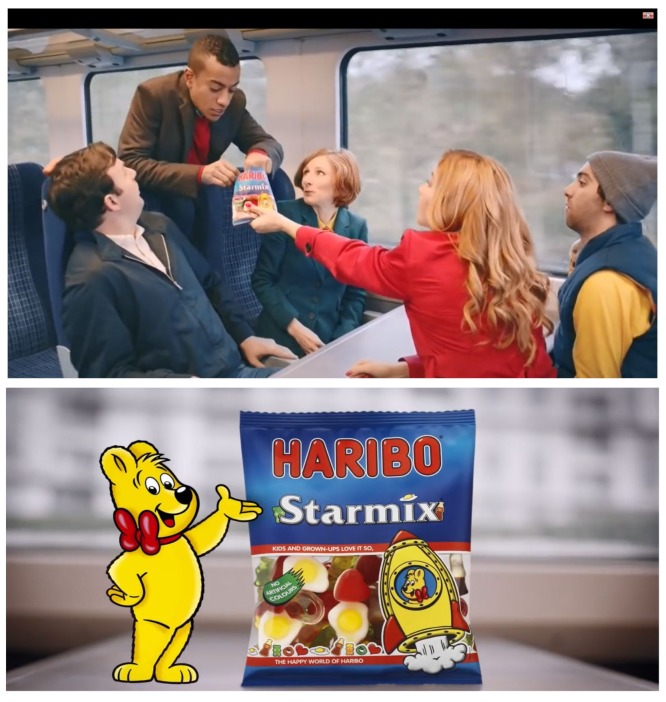
Still images from Haribo’s train advert (from https://www.youtube.com/watch?v=dSbJWsIScUE).

The second advert, from Haribo and lasting 30 s, represented a confectionery (sweets) brand that is affordable with ‘pocket money’. The advert is set on a busy commuter train, typical of the UK. The advert depicts a group of adults sat around a table on the train, with a bag of Haribo Starmix in the middle (Figure 2) [43]. There is a mixture of ages, genders, and ethnicities, all of whom are adults. All characters appear to be actors, rather than real-world celebrities. As they share sweets from the packet, the actors’ voices have been replaced with the voices of children saying what their favorite Haribo sweets are and why (e.g., “The egg is good, it’s all squidgy and soft”). The advert ends with the iconic Haribo jingle, “Kids and grown-ups love it so, in the happy world of Haribo”. With the exception of the jingle, there is no accompanying music, and the advert only features the ambient noise of train travel.

### 2.3. Measures

#### 2.3.1. Demographics

Details concerning gender, ethnicity, resident UK country (coded: England, Scotland, Wales, Northern Ireland), and a measure of deprivation (Index of Multiple Deprivation (IMD), a quantitative measure based on a respondent’s postcode and accounting for a variety of socio-demographic factors) were obtained from information held about respondents by YouGov or survey questions. We also obtained information on age (range: 11–19 years old). As the UK’s existing self-regulations define a child as someone under the age of 16 years old [32], we coded participants as being either 11–15 years or 16–19 years old. Ethnicity was binary coded for analysis purposes. As census data suggests that ‘White British’ is the dominant ethnicity in the UK ([44], also see Table 1 for weighted data), we dichotomized participants as ‘White British’ versus ‘Other ethnicities’, which is an approach that is consistent with previous reporting of this data [1,35]. 

#### 2.3.2. Body Mass Index

Participants were asked to self-report ‘How much do you weigh/How tall are you? Please be as accurate as possible’, with separate questions for each measure. For height, participants were presented with a response list that featured feet and inches (e.g., 5ft 4in) and the equivalents in centimeters (e.g., 163 cm). For weight, participants were presented with a response list that featured stones and pounds (e.g., 8st 11 lbs) and the equivalents in kilograms (e.g., 55.8 kgs) and pounds only (e.g., 123 lbs). Participants could also indicate ‘Prefer not to say’ and ‘Don’t Know’. Where possible, the BMI was calculated using the weight and height data, and participants were categorized using the extended International Obesity Task Force BMI classifications (including age and gender adjustments for 11–17 year olds) as either underweight, healthy weight, overweight, or obese [45].

#### 2.3.3. Reactions to the Adverts for HFSS Foods

Immediately after each advert was shown, participants were asked to confirm if they had been able to watch the content (Yes/No). If successfully watched, participants were asked to what extent each advert (1) Does not tempt me to try [product]/Tempts me to try the [product]; (2) Makes [product] seem unappealing/Makes [product] seem appealing; (3) Advert is fun/Advert is boring; (4) Made [product] seem unhealthy choice/Made [product] seem healthy choice; (5) Made [product] seem unpopular choice/Made [product] seem popular choice; (6) Made me think that having [product] is boring/Made me think that having [product] is fun; (7) Would be unappealing to people my age/Would be appealing to people my age; and (8) I dislike this advert/I like this advert. The reaction measures were based on preliminary focus group research with 11-19 year olds [16] and prior research exploring how adolescents react to marketing for other fast-moving consumer goods, such as tobacco [46]. For each reaction, responses were scored on a five-point Likert Scale (e.g., 1 = Made [product] seem unpopular choice to 5 = Made [product] seem popular choice). For each, the scale responses were binary coded into those which had a positive reaction (codes 4 and 5) and those which had a neutral or negative reaction (codes 1–3).

### 2.4. Analysis

Data were analysed using SPSS version 24 (SPSS Inc, Chicago, IL, USA). Weighted frequencies were used to examine sample demographics and the proportion of participants who had a positive reaction to each advert for each of the eight reaction measures. Binary logistic regressions were conducted with reactions to each advert as the respective dependent variables (e.g., positive reaction on appeal to age group for the Haribo advert, coded Yes/No). Participants who had not been able to watch either advert were excluded on a test-by-test basis. Covariates of age, gender, ethnicity, country, IMD quintile, and BMI category were included. Reference categories for the binary variables are reported in the results. For the IMD and BMI category, which had >3 more levels and were ordinal in nature, the contrast = difference function enabled a comparison of each increasing category relative to the combined preceding levels. For country, the contrast = simple function compared each of Scotland, Northern Ireland, and Wales to England.

### 2.5. Ethics

The study was approved by the University of Stirling’s General University Ethics Panel (GUEP59). Informed consent was obtained from all participants prior to taking part in the survey. All survey measures and study materials were designed based on preliminary focus group research [16] and were pilot tested for cultural and age appropriateness with the target sample.

## 3. Results

### 3.1. Sample Characteristics

The weighted sample (*n* = 3348) had an average age of 15.15 years (SD = 2.56), with 53% aged 11–15 years old and 47% aged 16–19 years old (Table 1). There was an approximately even distribution of males (51%) and females (49%). The majority of respondents were white British (76%) and lived in England (84%). There was an even proportion from each of the quintiles of deprivation (20%) (Table 1). After excluding participants with missing data for height or weight status (*n* = 816, weighted), the majority of the weighted sample (62%) had a BMI categorized as healthy weight. Seventeen percent of participants had a BMI classed as underweight, 16% as overweight, and 5% as obese. 

### 3.2. Reactions to the Fast-Food Advert

Three quarters of adolescents (72%, weighted) thought that the fast-food advert made McDonald’s appear a popular choice (Table 2). Around half thought that the fast-food advert would appeal to people their age (56%), made McDonald’s seem fun (55%) or appealing (50%), and that the advert was fun (51%). Approximately a third reported liking the fast-food advert overall (32%) and that it tempted them to try McDonalds (31%). Around a quarter thought that the advert made McDonald’s seem a healthy choice (28%). 

Binary logistic regression found that younger adolescents (i.e., 11–15 year olds) were more likely to report that the fast-food advert would appeal to their age group (*Adjusted Odds Ratio* (*AOR*) = 1.33, 95% CI: 1.13–1.58), that they liked the advert (*AOR* = 1.32, 95% CI: 1.11–1.58), and that the advert tempted them to try McDonalds (*AOR* = 1.54, 95% CI: 1.28–1.84) (Table 3). Younger adolescents were less likely to report that the fast-food advert made McDonald’s appear a popular choice (*AOR* = 0.63, 95% CI: 0.52–0.77). Females were more likely than males to have positive reactions for seven of the eight measures; the exception was reporting that the advert made McDonald’s appear healthy, which had no association with gender (*p* = 0.93). There was a main effect of IMD on temptation to try, with those from the third (*AOR* = 0.79, 95% CI: 0.62–0.99) and fourth (*AOR* = 0.64, 95% CI: 0.52–0.81) IMD quintiles being less likely to report temptation to try than those from more deprived categories. There was a main association between BMI and perceived product popularity (*p* = 0.035), although there were no significant associations within individual category comparisons. There was no main association of BMI for all other reactions, although there were two associations to acknowledge within comparisons of the individual BMI levels. Specifically, those with an obese BMI were more likely than other (lower) BMI groups to report that the fast-food advert tempted them to try McDonald’s (*AOR* = 1.53, 95% CI: 1.05–2.25), while those with an overweight BMI were more likely than those with a healthy or underweight BMI to report that the advert made McDonald’s seem fun (*AOR* = 1.32, 95% CI: 1.04–1.67).

### 3.3. Reactions to the Confectionery Advert

Approximately three quarters of adolescents (71%, weighted) thought that the confectionery advert made Haribo appear a popular choice (Table 4). Around two thirds of adolescents also thought that the advert made the Haribo product seem fun (64%) and that the advert was fun (68%). Approximately half of adolescents also said they thought that the advert made Haribo look appealing (56%), that they liked the confectionery advert overall (56%), and that they thought it would appeal to people their age (48%). Two-fifths (41%) said that the advert tempted them to try Haribo. One in ten adolescents (10%) thought that the advert made Haribo appear a healthy choice.

Binary logistic regressions found that younger adolescents (i.e., 11–15 year olds) were more likely to report that the confectionery advert would appeal to their age group (*AOR* = 1.79, 95% CI: 1.51–2.11), that they liked the advert (*AOR* = 1.19, 95% CI: 1.01–1.41), that it made Haribo appear a healthy choice (*AOR* = 2.20, 95% CI: 1.64–1.96), and that the advert tempted them to try Haribo (*AOR* = 1.57, 95% CI: 1.32–1.86) (Table 5). Younger adolescents were less likely to report that the confectionery advert made the product appear popular (*AOR* = 0.74, 95% CI: 0.61–0.89). Concerning gender, females were more likely than males to have positive reactions to the advert for seven of the eight measures; the exception was reporting that the advert made Haribo appear a healthy option, which had no association with gender (*p* = 0.52). Concerning BMI, there was only a main association for product appeal (*p* = 0.028); adolescents who had an overweight BMI were more likely than those with a healthy or underweight BMI to report that the advert made Haribo appear appealing (*AOR* = 1.38, 95% CI: 1.09–1.74). There was no main association of BMI for all other reactions, although there were two associations to acknowledge within comparisons of the individual BMI levels. Specifically, adolescents with an obese BMI were more likely than other BMI groups to report that the confectionery advert would appeal to their age group (*AOR* = 1.54, 95% CI: 1.06–2.34) and that it made Haribo appear a popular choice (*AOR* = 1.73, 95% CI: 1.08–2.76). 

## 4. Discussion

The principal finding is that adolescents in the UK reacted positively to the two HFSS food adverts, with the majority having positive reactions on five of the eight measures for each advert. This included positive perceptions concerning the brand (e.g., perceived popularity and appeal), advert design (e.g., fun and appeal to age group), and impact on behavior (e.g., temptation to try). A secondary finding is that positive reactions had key associations with demography and, to a lesser extent, BMI category. This included that younger adolescents were more likely to report that both adverts would appeal to their age group and that they were tempted to try the brands promoted.

The findings are consistent with consumer research that has demonstrated the importance of examining how marketing shapes and reinforces attitudes towards HFSS products/brands [7,8,9,10,11,12,13,14,15,16,17,18,19,20,21], in addition to research examining the associations between the volume and frequency of marketing exposure and dietary outcomes [1]. The findings are also consistent with suggestions that some young people may be more susceptible to the persuasive intent of HFSS marketing. For example, younger adolescents were more likely to report positive reactions to the two adverts for several measures, including perceived appeal to their age group, temptation to try, and perceived healthiness of the confectionery product. This trend may be explained, at least partly, by suggestions that the ability of younger age groups to recognize the commercial and persuasive intent of marketing is less developed and, therefore, they may be more susceptible [47,48]. Gender was also an important factor, with females more likely than males to react positively for all measures for both adverts, except perceived healthiness. Compared to other fast-moving consumer goods, such as tobacco [49,50] and alcohol [51], there is comparatively less research on the role of gender in the design and impact of HFSS marketing [52]. This represents an important avenue for future research, particularly using in-depth qualitative methodologies that continue to be under-represented in the HFSS marketing literature [5]. For the most part, there was no main association between the BMI category and positive advert reactions. Comparisons of individual categories, however, did suggest some tentative associations. For example, those with an obese BMI were more likely to report temptation to try the fast-food product and perceived age appeal for the confectionery brand. This study, however, was only based on self-reported BMI, which may not always provide accurate estimations among young people [53]. Further research based on anthropometric measures (e.g., in-person experiments) could better explore to what extent (if at all) the BMI category is associated with positive reactions and susceptibility. 

### 4.1. Potential Avenues for Future Research and Potential Implications for Regulation

If the results of this study are typical of reactions to wider marketing activities for HFSS food products/brands in the UK, then our findings highlight three avenues for future research and regulation change that may offer better protection to children and adolescents. First, a quarter of adolescents thought that the fast-food advert made the brand appear healthy and one in ten thought this for the confectionery advert; 11–15 year olds were two times more likely than older adolescents to think that the confectionery advert made the product appear healthy. Although neither advert explicitly claimed that their products were healthy or nutritious, neither contained any information to the contrary either; a trend consistent with previous content analysis research on the marketing of HFSS foods [21]. In the fast-food advert, it could also be argued that healthiness was passively/indirectly implied through the activities depicted (e.g., playing football and running on a beach) and the products clearly shown (e.g., bottles of water or fruit juice—the only time an HFSS product is clearly visible is on a waterproof top, see top right image in Figure 1). In France, all television adverts for HFSS foods must display one of four health messages and, from January 2021, it will also become mandatory to include a ‘nutri-score’ graphic that provides an easy-to-read indication of the nutritional content [31,54]. Evaluating what impact, if any, nutri-scores and mandatory health messages in advertising have on consumer perceptions of HFSS product healthiness, or whether knowing the nutritional content would have had a moderating effect on other advert reactions in this study (e.g., temptation to try and perceived popularity), represents an important avenue for research that could inform regulatory change in the UK.

Second, for both adverts, younger adolescents (i.e., 11–15 year olds) were more likely to report several positive reactions than older adolescents (i.e., 16–19 year olds), including appeal to their age group and temptation to try the products/brands depicted. In the UK, there is incongruence in how appeal to young people is defined across different parts of the same self-regulatory codes. For HFSS foods, marketing must not be directed at those aged under 16 years (i.e., children) through either the selection of media or the context in which it appears [32]. There are also restrictions on using licensed characters (e.g., celebrities) if targeting consumers under 11 years old. Elsewhere in the same self-regulations, however, appeal to young people is defined differently. For instance, marketing for age-restricted products such as alcohol, gambling, and electronic cigarettes must not particularly appeal to those under 18 years old (e.g., by creating associations with youth culture) and must not prominently feature real or fictitious people who are (or appear to be) under 25 years old [55,56,57]. These stipulations are in addition to placement restrictions similar to HFSS foods. The two adverts in this study did plausibly contain content that would not have been permitted under these alternate definitions, including associations with youth culture (e.g., the pop-group ‘Little Mix’), content that may appeal to young people (e.g., humorous child-like voiceovers), and featuring young people under 25 years old. Future research should therefore explore to what extent, if at all, applying these alternate regulatory criteria to the marketing of HFSS foods could reduce appeal to children and younger adolescents. Research could also further consider how both approaches compare to other more restrictive statutory controls on marketing design and content. For example, alcohol advertising in France is only permitted to include factual information about the products depicted; no lifestyle and evocative messages are allowed, such as those which feature in the creatives used in this study [31,58].

Third, the findings show the potential benefit of involving the relative expertise of young people in interpreting and highlighting the appeal of advertising among this age group—something seemingly lacking in the current self-regulatory framework. Previous research has shown that the adjudications of self-regulatory bodies—including whether marketing may appeal to young people—are not always consistent with the views of harm-reduction and advocacy organisations [34,59], researchers [60], the general population [61], or young people [62]. To date, however, much of this research has focused on other age-restricted fast-moving consumer goods, in particular alcohol. Future studies should therefore use these existing methodologies to examine to what extent, if at all, existing self-regulatory decisions for HFSS marketing are congruent to the perspectives of young people. Where incongruence is evident, research exploring the perspectives of multiple stakeholders involved in the production, research, regulation, and consumption of marketing—including children, adolescents, and their parents—would be of value to identify effective ways of reducing appeal and to form a consensus on effective and appropriate regulatory change. 

### 4.2. Limitations

The Youth Obesity Policy Survey covered a variety of topics [1,35,38,39,40] and, due to space restraints, we only included two adverts for HFSS foods as stimuli. These adverts were intended to be illustrative, but not representative, of all HFSS products; reactions may vary for other brands or other marketing creatives from the two brands featured (e.g., different marketing campaigns). Replication across different HFSS products and brands is imperative. We also did not include a comparator advert, to ascertain to what extent (if at all) reactions to HFSS food marketing differs to advertising for non-food products (e.g., toys or electronic devices). The creatives also only represent video adverts suitable for television or online placement, and are not representative of all marketing activities (e.g., print, packaging, or sponsorship). The eight reaction measures, albeit informed by focus groups [16] and existing research [46], are not exhaustive of all possible reactions. It is plausible that young people may have formed other opinions to the adverts (both positive and negative) that were not captured in the measures, and a more detailed understanding concerning appeal and interpretation could have been generated by showing creatives in qualitative research [15,16,17,18,19,20]. We also cannot determine to what extent, if at all, reactions were influenced by existing heuristics and attitudes towards the brands depicted (e.g., due to prior exposure to other forms of marketing). Artificial exposure in a survey and self-reporting reactions is not representative of real-world exposure to advertising, where processing may be automatic or there may be other competing attentional burdens. Finally, the findings only show reactions to the adverts and self-reported temptation to try. The data cannot show whether the adolescents would have acted on the promotional messages or any subsequent effect on weight. 

## 5. Conclusions

Adolescents in the UK reported a variety of positive reactions to the two HFSS adverts shown in this study, including perceived brand popularity, age appeal, perceived healthiness, and temptation to try. There were key associations with demography and, to a lesser extent, BMI category, which suggests that some adolescents are more susceptible to the influence of advertising for HFSS foods. For example, younger adolescents were more likely to report perceived appeal to their age group and temptation to try, while female adolescents were more likely to have positive reactions for seven of the eight measures. If these reactions are typical of marketing for other HFSS food products and brands, the findings link to three possible avenues for research that could help improve the efficacy of regulation in the UK and offer better protection to children and young people. These include testing the impact of clearly displaying nutritional information and health messaging in adverts and testing alternative methods of regulating appeal to young people based on existing approaches for other fast-moving consumer goods (e.g., age-restricted products). Additionally, they include testing to what extent, if at all, existing self-regulatory decisions are congruent to the views of young people, and involving the perspectives of multiple stakeholders (including children and their parents) in identifying effective and appropriate revisions to regulation. 

## Figures and Tables

**Table 1:** Sample profile based on unweighted and weighted frequencies.

	Unweighted		Weighted	
**Variable**	%	*n*	%	*n*
**Age Group**				
11–15 years old	60	2010	53	1774
16–19 years old	40	1338	47	1574
**Gender**				
Male	48	1596	51	1707
Female	52	1752	49	1641
Ethnicity				
White British	84	2810	76	2555
Other	16	520	23	775
Not specified or prefer not to say	<1	18	<1	17
**Country Lived In**				
England	76	2534	84	2826
Scotland	13	419	8	261
Wales	8	251	5	157
Northern Ireland	4	144	3	104
**IMD Quintile**				
1 (most deprived)	16	534	20	670
2	21	695	20	670
3	22	731	20	670
4	24	787	20	670
5 (least deprived)	18	601	20	670
**Weight Status ⸸** **Δ**				
Underweight	17	412	17	438
Healthy weight	62	1549	62	1556
Overweight	16	395	16	411
Obese	5	129	5	127

Base: All participants; ⸸ based on the Extended International (IOTF) Body Mass Index Classification, including age and gender adjustments for 11–17 year olds; ^Δ^ missing data due to missing height or weight information (n = 816, weighted)

**Table 2:** Positive reactions to the fast-food advert.

	Overall Sample		11–15 Year Olds	
Reaction to Fast-Food Advert	%	*n*	%	*n*
Made product seem popular choice	72	2335	69	1186
Appealing to age group	56	1795	59	1010
Made product seem fun	55	1772	55	943
Advert was fun	51	1651	50	869
Made product look appealing	50	1620	49	846
Liked the advert	32	1024	34	590
Tempted them to try product	31	1004	35	605
Made product seem a healthy choice	28	900	27	464

Base = All participants; data are weighted; missing data on those who could not watch advert (*n* = 119, weighted); variables binary coded from a five-point scale into positive versus neutral and negative reactions.

**Table 3:** Reactions to the fast-food advert and associations with demography and the BMI group.

Reactions to the Fast-Food (McDonald’s) Advert																	
		Seemed Popular		Age Appeal		Product Fun		Advert Fun		Product Appealing		Liked Advert		Product Healthy		Temptation to Try	
**Variables**	*n*	*AOR*	*p*	*AOR*	*p*	*AOR*	*p*	*AOR*	*p*	*AOR*	*p*	*AOR*	*p*	*AOR*	*P*	*AOR*	*p*
**Age**																	
16–19 years old	1323	Ref	-	Ref	-	Ref	-	Ref	-	Ref	-	Ref	-	Ref	-	Ref	-
11–15 years old	1063	0.63	0.001	1.33	0.001	0.96	0.65	0.95	0.52	0.88	0.13	1.32	0.002	0.94	0.49	1.54	0.001
**Gender**																	
Male	1139	Ref	-	Ref	-	Ref	-	Ref	-	Ref	-	Ref	-	Ref	-	Ref	-
Female	1247	1.45	0.001	1.34	0.001	1.59	0.001	1.82	0.001	1.42	0.001	1.50	0.001	1.01	0.93	1.24	0.02
**Ethnicity**																	
Other	397	Ref	-	Ref	-	Ref	-	Ref	-	Ref	-	Ref	-	Ref	-	Ref	-
White British	1989	1.19	0.17	1.16	0.19	1.01	0.97	0.96	0.73	0.97	0.79	0.91	0.45	0.95	0.70	1.09	0.48
**Country**			0.87		0.42		0.75		0.44		0.55		0.80		0.10		0.41
England	1808	Ref	-	Ref	-	Ref	-	Ref	-	Ref	-	Ref	-	Ref	-	Ref	-
Wales (*vs. England*)	184	1.09	0.63	1.29	0.12	1.19	0.28	1.08	0.63	1.02	0.90	1.01	0.96	0.92	0.62	1.10	0.58
Scotland (*vs. England*)	275	0.92	0.56	0.95	0.69	0.99	0.95	1.13	0.36	1.13	0.35	1.03	0.81	1.36	0.03	1.20	0.19
N. Ireland (*vs. England*)	119	1.04	0.86	1.08	0.68	1.02	0.93	1.32	0.16	1.26	0.24	1.22	0.33	1.25	0.28	1.26	0.25
**IMD**			0.30		0.31		0.33		0.41		0.75		0.36		0.08		0.001
1	397	Ref	-	Ref	-	Ref	-	Ref	-	Ref	-	Ref	-	Ref	-	Ref	-
2 *(vs. 1)*	490	0.91	0.54	1.02	0.89	0.99	0.92	1.04	0.78	0.91	0.49	0.97	0.85	1.13	0.39	0.86	0.27
3 *(vs. 1, 2)*	514	1.31	0.04	1.12	0.34	0.97	0.80	1.05	0.96	0.96	0.68	1.11	0.39	0.84	0.15	0.79	0.04
4 *(vs. 1, 2, 3)*	570	1.00	0.98	0.82	0.06	0.81	0.04	0.89	0.27	0.89	0.25	0.82	0.06	0.89	0.32	0.64	0.001
5 *(vs. 1, 2, 3, 4)*	415	0.96	0.74	1.03	0.80	1.05	0.68	1.19	0.12	1.02	0.86	1.00	0.98	0.77	0.03	0.94	0.62
**Weight Status**			0.04		0.27		0.06		0.51		0.15		0.13		0.23		0.13
Underweight	397	Ref	-	Ref	-	Ref	-	Ref	-	Ref	-	Ref	-	Ref	-	Ref	-
Healthy weight *(vs. u/w^4^)*	1490	1.25	0.07	1.14	0.24	1.12	0.34	1.07	0.56	1.17	0.16	1.08	0.52	1.27	0.07	1.01	0.95
Overweight *(vs. u/w & h’lthy)*	375	1.26	0.09	1.23	0.09	1.32	0.02	1.11	0.37	1.23	0.08	1.17	0.21	0.99	0.95	1.09	0.53
Obese *(vs. all other)*	124	1.61	0.05	1.15	0.46	1.30	0.18	1.25	0.24	1.25	0.24	1.47	0.05	1.24	0.29	1.53	0.03

Notes: Dependent variable for all models: did the participant have a positive reaction (codes 4/5) or a neutral and negative reaction (codes 1–3); Hosmer and Lemeshow for all models *p* > 0.05; *AOR* = Adjusted Odds Ratio; cases with missing data on one or more variables in all models (*n* = 962, i.e., could not watch video or report BMI).

**Table 4:** Positive reactions to the confectionery advert.

	Overall Sample		11–15 Year Olds	
Reaction to Fast-Food Advert	%	*n*	%	*n*
Made product seem popular choice	71	2328	69	1210
Advert was fun	68	2229	69	1200
Made product seem fun	64	2089	64	1119
Made product look appealing	56	1835	58	1011
Liked the advert	56	1826	59	1036
Appealing to age group	48	1572	55	963
Tempted them to try product	41	1329	46	810
Made product seem a healthy choice	10	330	13	226

Base = All participants; data are weighted; missing data on those who could not watch advert (*n* = 74, weighted); variables binary coded from a five-point scale into positive versus neutral and negative reactions.

**Table 5:** Reactions to the confectionery (Haribo) advert and associations with demography and the BMI group.

		Reactions to the Confectionery (Haribo) Advert															
		Seemed Popular		Age Appeal		Product Fun		Advert Fun		Product Appealing		Liked Advert		Product Healthy		Temptation to Try	
**Variables**	*n*	*AOR*	*p*	*AOR*	*p*	*AOR*	*p*	*AOR*	*p*	*AOR*	*p*	*AOR*	*p*	*AOR*	*P*	*AOR*	*p*
**Age**																	
16–19 years old	1341	Ref	-	Ref	-	Ref	-	Ref	-	Ref	-	Ref	-	Ref	-	Ref	-
11–15 years old	1075	0.74	0.001	1.79	0.001	0.98	0.77	0.92	0.38	1.09	0.32	1.19	0.04	2.20	0.001	1.57	0.001
**Gender**																	
Male	1146	Ref	-	Ref	-	Ref	-	Ref	-	Ref	-	Ref	-	Ref	-	Ref	-
Female	1270	1.36	0.001	1.18	0.04	1.33	0.001	1.21	0.03	1.39	0.001	1.40	0.001	0.92	0.52	1.27	0.004
**Ethnicity**																	
Other	404	Ref	-	Ref	-	Ref	-	Ref	-	Ref	-	Ref	-	Ref	-	Ref	-
White British	2012	1.07	0.57	1.12	0.32	1.00	0.99	1.06	0.64	1.13	0.27	1.32	0.01	0.71	0.05	0.97	0.81
**Country**			0.13		0.17		0.43		0.06		0.16		0.01		0.06		0.16
England	1829	Ref	-	Ref	-	Ref	-	Ref	-	Ref	-	Ref	-	Ref	-	Ref	-
Wales (*vs. England*)	186	1.23	0.26	1.17	0.31	1.05	0.77	1.12	0.51	1.19	0.27	0.91	0.56	1.21	0.45	1.30	0.10
Scotland (*vs. England*)	281	0.79	0.09	0.78	0.06	0.82	0.12	0.72	0.01	0.79	0.07	0.65	0.001	0.87	0.55	0.95	0.71
N. Ireland (*vs. England*)	120	1.24	0.34	0.95	0.80	1.06	0.76	0.92	0.70	1.03	0.87	1.00	0.98	1.88	0.01	1.35	0.12
**IMD**			0.55		0.44		0.08		0.40		0.72		0.41		0.17		0.19
1	405	Ref	-	Ref	-	Ref	-	Ref	-	Ref	-	Ref	-	Ref	-	Ref	-
2 *(vs. 1)*	495	0.83	0.20	0.86	0.26	0.80	0.10	0.93	0.59	0.86	0.27	0.80	0.11	0.96	0.84	0.97	0.80
3 *(vs. 1, 2)*	522	1.12	0.37	1.04	0.73	1.30	0.02	1.24	0.08	0.99	0.91	1.11	0.37	0.74	0.10	0.93	0.51
4 *(vs. 1, 2, 3)*	571	1.02	0.90	0.86	0.12	1.01	0.95	0.94	0.57	0.95	0.58	0.95	0.59	0.71	0.06	1.04	0.70
5 *(vs. 1, 2, 3, 4)*	423	1.07	0.58	0.99	0.92	0.97	0.79	1.05	0.70	0.91	0.40	1.04	0.72	0.99	0.95	0.77	0.02
**Weight Status**			0.10		0.05		0.15		0.29		0.03		0.14		0.56		0.96
Underweight	396	Ref	-	Ref	-	Ref	-	Ref	-	Ref	-	Ref	-	Ref	-	Ref	-
Healthy *(vs. u/w^4^)*	1510	1.15	0.25	0.96	0.74	1.01	0.93	1.04	0.76	1.17	0.18	0.89	0.32	1.06	0.78	1.01	0.93
Overweight *(vs. u/w & h’lthy)*	383	1.08	0.55	1.14	0.28	1.20	0.14	1.03	0.80	1.38	0.01	1.12	0.35	1.19	0.35	0.96	0.75
Obese *(vs. all other)*	127	1.73	0.02	1.54	0.02	1.40	0.12	1.51	0.06	1.27	0.22	1.32	0.15	1.36	0.30	1.09	0.64

Notes: Dependent variable for all models: did the participant have a positive reaction (codes 4/5) or a neutral and negative reaction (codes 1–3); Hosmer and Lemeshow for all models *p* > 0.05, except product fun, where *χ^2^*(8) = 18.75, *p* = 0.016; *AOR* = Adjusted Odds Ratio; cases with missing data on one or more variables in all models (*n* = 932, i.e., could not watch video or report BMI).
