# Correction: Critchlow, N., et al. Adolescents’ Reactions to Adverts for Fast-Food and Confectionery Brands That Are High in Fat, Salt, and/or Sugar (HFSS), and Possible Implications for Future Research and Regulation: Findings from a Cross-Sectional Survey of 11–19 Year Olds in the United Kingdom. *Int. J. Environ. Res. Public Health* 2020, *17*, 1689

**DOI:** 10.3390/ijerph18063181

**Published:** 2021-03-19

**Authors:** Nathan Critchlow, Jessica Newberry Le Vay, Anne Marie MacKintosh, Lucie Hooper, Christopher Thomas, Jyotsna Vohra

**Affiliations:** 1 Institute for Social Marketing and Health, Faculty of Health Sciences and Sport, University of Stirling, Stirling FK9 4LA, UK; a.m.mackintosh@stir.ac.uk; 2 Cancer Policy Research Centre, Cancer Research UK, 2 Redman Place, London E20 1JQ, UK; jessica.newberrylevay@cancer.org.uk (J.N.L.V.); lucie.hooper@cancer.org.uk (L.H.); chris.thomas@cancer.org.uk (C.T.); jyotsna.vohra@cancer.org.uk (J.V.)

## 1. Introduction

It has come to our attention that there are two errors in our recent article [1]. First, the extended International Obesity Task Force (IOTF) classifications [2]—including age and gender adjustments for 11–17-year-olds—were used to categorize participants’ Body Mass Index (BMI) as underweight, healthy weight, overweight, or obese. We have determined, however, that a minority of female participants aged 11–17 years old were accidentally assigned the incorrect IOTF grade for their BMI. Second, while investigating this, it was found that a miscommunication/misunderstanding within the research team also necessitates two minor clarifications to the methods.

Hereafter, this correction provides a summary of how the IOTF grade misclassification was detected by the authors, the remedial action taken, and the changes to the original manuscript. The authors note that while the correction to some IOTF grades has necessitated some revisions to the original results—including some new associations between weight status and advert reactions—the overall scientific conclusions are unaffected. The original manuscript has been updated and the authors apologize for any inconvenience.

## 2. Explaining Changes to the IOTF Grading

### 2.1. What Does the Correction Relate to?

For extended IOTF grading, the BMI thresholds used to determine whether an individual is considered underweight, healthy weight, overweight or obese vary by sex for those aged ≤17 years old [2]; the BMI thresholds are the same for males and females aged ≥18 years. It has come to our attention, however, that the IOTF grading for participants aged 11–17 years old in the final dataset received by the research team was unintentionally only based on the BMI thresholds for males, and therefore a minority of female participants were assigned the wrong extended IOTF grade.

### 2.2. How Was This Detected?

The data come from a project called the Youth Obesity Policy Survey (YOPS), which is commissioned and overseen by Cancer Research UK (CRUK). The project is based on a repeat cross-sectional design, with waves conducted in 2017 and 2019. The fieldwork for both waves was conducted by YouGov, a market research company. While the dataset for the 2017 YOPS—which the original manuscript is based on—already contained an IOTF grading variable on delivery to CRUK, the latter 2019 wave did not. When CRUK then commissioned analyses of the latter dataset, this meant that the corresponding author (N.C.) needed to compute IOTF grading for the 2019 YOPS wave. However, when the two datasets were combined to analyse trends over time, N.C. detected that the BMI thresholds for IOTF grading were inconsistent between waves. Following investigation, N.C. determined that all instances of misclassification related to female participants aged 11–17 years old in the 2017 YOPS wave. Put simply, while the male BMI data from the 2017 YOPS matched the relevant extended IOTF thresholds [2], the only way the female BMI data matched the grade assigned was if compared to the male BMI thresholds.

### 2.3. How Much Data Does This Concern?

Based on the four IOTF grades reported in the manuscript (underweight, healthy weight, overweight, and obese), only a minority of female participants aged 11–17 years old were assigned the wrong IOTF grade. The data for male participants are unaffected, as too are data for females aged ≥18 years (the extended IOTF thresholds are the same for adults irrespective of gender), and the participants who did not provide valid height/weight data to compute BMI and, therefore, an IOTF grade. In total, we estimate that <2% of the sample were assigned the incorrect IOTF grade. As part of our investigations, we also detected one additional participant who was erroneously marked as ‘missing data’ for IOTF grade, but for whom there was valid BMI data to assign grading.

### 2.4. Remedial Action Taken

We have recalibrated the data so the extended IOTF classifications for the minority of impacted female participants aged 11–17 years old are now correct. We have then re-computed the logistic regression models to incorporate both the revised IOTF grading and the additional participant with a newly assigned BMI; those with a ‘not stated’ BMI were excluded from the original regression models. We have also made two minor clarifications to the methods. The outcomes of these changes are detailed below:

## 3. The Corrections

### 3.1. Change to the Abstract

The 95% Confidence Intervals (CI) concerning the Adjusted Odds Ratios (OR) for the confectionery advert have been updated to reflect revisions elsewhere in the results/manuscript. The revisions to the values are minor, and the direction of effect and statistical significance is unchanged. The updated text reads:

“*For example, 11–15 year olds were more likely than 16–19 year olds to report appeal to their age group for the fast-food (OR = 1.33, 95% CI: 1.13–1.58) and confectionery advert (OR = 1.79, 95% CI: 1.52–2.12)*.”

### 3.2. Change to 2.1. Design and Recruitment

In the methods, updated text clarifies that it was area level of deprivation, not social grade, that was used in the survey weighting:

“*A survey weight enabled descriptive data to be representative of the UK population (based on age, gender, ethnicity, region, and area level of deprivation)*.”

### 3.3. Change to 2.3.2. Body Mass Index

In the methods, updated text clarifies that, for participants aged 11–15 years old, height and weight data was reported by parent(s)/guardian(s), not participants:

“*Participants were asked to self-report ‘How much do you weigh/How tall are you? Please be as accurate as possible’, with separate questions for each measure. This data was self-reported by the participants aged 16–19 years old, and by parent(s)/guardian(s) for 11–15 year olds*.”

### 3.4. Change to 3.1. Sample Characteristics

The original manuscript stated that 62% of the (weighted) sample had a BMI classified as healthy weight, 17% as underweight, 16% as overweight and 5% as obese. The percentage values for healthy weight and obese are unchanged by the updated IOTF grading, as is the weighted amount of missing data, but updated text provides revised percentage values for the underweight and overweight categories:

“*After excluding participants with missing data for height or weight status (n = 816, weighted), the majority of the weighted sample (62%) had a BMI categorized as healthy weight. Eighteen percent had a BMI classed as underweight, 15% as overweight, and 5% as obese*.”

The percentage and nominal values for weight status in Table 1 are also updated in line with the above. A revised Table 1 is provided at the end of this correction. The values for age, gender, ethnicity, country lived in, and IMD quintile are unchanged.

### 3.5. Change to 3.2. Reactions to the Fast-Food Advert

As a consequence of the re-calibrated IOTF grading, the logistic regressions now show three associations between weight status and reactions to the fast-food advert that were not statistically significant previously: (1) participants with an obese BMI were more likely than other BMI groups to perceive that the fast food advert made McDonald’s seem popular; (2) participants with an obese BMI were more likely than other BMI groups to say they liked the fast-food advert; and (3) participants with overweight BMI were more likely than those with a healthy or underweight BMI to perceive that the fast food advert made McDonald’s seem appealing. In the revised analyses, there was also a new omnibus association between weight status and perceived product fun, albeit the individual-level association between overweight BMI and perceived product fun was already reported in the text. The other associations with weight status and reactions to the fast-food advert are unchanged.

Updated text for the logistic regression results is provided below, and an updated version of Table 3 is provided at the end of this correction. Please note, that as a consequence of re-running the regressions, the participant ns, ORs, 95% CIs, and *p* values for other covariates display minor changes but, with the exception of those described, there were no other changes in direction of effect or statistical significance to the results reported:

“*Binary logistic regressions found that younger adolescents (i.e., 11–15 year olds) were more likely to report that the fast-food advert would appeal to their age group (Adjusted Odds Ratio (AOR) = 1.33, 95% CI: 1.13–1.58), that they liked the advert (AOR = 1.33, 95% CI: 1.11–1.59), and that the advert tempted them to try McDonalds (AOR = 1.53, 95% CI: 1.28–1.84) (Table 3). Younger adolescents were less likely to report that the fast-food advert made McDonald’s appear a popular choice (AOR = 0.64, 95% CI: 0.53–0.77). Females were more likely than males to have positive reactions for seven of the eight measures; the exception was reporting that the advert made McDonald’s appear healthy, which had no association with gender (p = 0.94). There was a main effect of IMD on temptation to try, with those from the third (AOR = 0.79, 95% CI: 0.62–0.99) and fourth (AOR = 0.64, 95% CI: 0.51–0.80) IMD quintile being less likely to report temptation to try than those from more deprived categories*.

*The binary logistic regressions showed main associations between BMI and perceived popularity (p = 0.02) and product fun (p = 0.03), including those with an obese BMI being more likely than other weight groups to perceive the fast food advert made McDonald’s appear popular (AOR = 1.70, 95% CI: 1.03–2.80) and those with an overweight BMI more likely than those with a healthy or underweight BMI to report that the advert made McDonald’s seem fun (AOR = 1.33, 95% CI: 1.04–1.69). There was no main association of BMI for all other reactions, although there were three associations to acknowledge within comparisons of the BMI levels. Specifically, those with an obese BMI were more likely than lower BMI groups to report that the fast-food advert tempted them to try McDonald’s (AOR = 1.56, 95% CI: 1.05–2.30) and that they liked the advert (AOR = 1.53, 95% CI: 1.04–2.25), while those with an overweight BMI were more likely than those with a healthy or underweight BMI to perceive that the advert made McDonald’s seem appealing (AOR = 1.29, 95% CI: 1.01–1.63)*.”

### 3.6. Change to 3.3. Reactions to the Confectionery Advert

As a consequence of the re-calibrated IOTF grading, the logistic regressions show one association between weight status and reactions to the confectionery advert that was not statistically significant previously: specifically, those with an obese BMI were more likely than other weight groups to report that the confectionary advert was fun. In the revised analysis, there was also a new omnibus association between weight status and perceived appeal to age group, albeit the individual association between having an obese BMI and perceived age appeal was already reported.

Updated text for the logistic regression results is provided below, and an updated version of Table 5 is provided at the end of this manuscript. Please note that as a consequence of re-running the regression models, the participant ns, ORs, 95% CIs, and *p* values for other covariates display minor changes but, with the exception of those described, there were no other changes in direction of effect or statistical significance in the results reported:

“*Binary logistic regressions found that younger adolescents (i.e., 11–15 year olds) were more likely to report that the confectionery advert would appeal to their age group (AOR = 1.79, 95% CI: 1.52–2.12), that they liked the advert (AOR = 1.20, 95% CI: 1.01–1.42), that it made Haribo appear a healthy choice (AOR = 2.22, 95% CI: 1.65–2.99), and that the advert tempted them to try Haribo (AOR = 1.57, 95% CI: 1.33–1.86) (Table 5). Younger adolescents were less likely to report that the confectionery advert made the product appear popular (AOR = 0.74, 95% CI: 0.61–0.89). Concerning gender, females were more likely than males to have positive reactions to the advert for seven of the eight measures; the exception was reporting that the advert made Haribo appear a healthy option, which had no association with gender (p = 0.55). Concerning BMI, there was a main association for perceived product appeal (p = 0.03) and appeal to age group (p = 0.02). Specifically, adolescents with an overweight BMI were more likely than those with a healthy or underweight BMI to report the advert made Haribo appear appealing (AOR = 1.37, 95% CI: 1.08–1.75), and those with an obese BMI were more likely than other weight groups to report appeal to their age group (AOR = 1.72, 95% CI: 1.17–2.53). There was no main association of BMI for all other reactions, although there were two associations to acknowledge within comparisons of the individual BMI levels. Specifically, adolescents with an obese BMI were more likely than other weight groups to report that the confectionery advert made Haribo appear a popular choice (AOR = 1.78, 95% CI: 1.09–2.90) and that the advert was fun (AOR = 1.67, 95% CI: 1.07–2.63)*.”

**Table 1:** Sample profile based on unweighted and weighted frequencies.

	Unweighted		Weighted	
Variable	%	*n*	%	*n*
**Age Group**				
11–15 years old	60	2010	53	1774
16–19 years old	40	1338	47	1574
**Gender**				
Male	48	1596	51	1707
Female	52	1752	49	1641
**Ethnicity**				
White British	84	2810	76	2555
Other	16	520	23	775
Not specified or prefer not to say	<1	18	<1	17
**Country Lived In**				
England	76	2534	84	2826
Scotland	13	419	8	261
Wales	8	251	5	157
Northern Ireland	4	144	3	104
**IMD Quintile**				
1 (most deprived)	16	534	20	670
2	21	695	20	670
3	22	731	20	670
4	24	787	20	670
5 (least deprived)	18	601	20	670
**Weight Status ^†,Δ^**				
Underweight	17	431	18	456
Healthy weight	63	1563	62	1568
Overweight	15	371	15	387
Obese	5	121	5	121

Base: All participants; ^†^ based on the extended International (IOTF) Body Mass Index Classification, including age and gender adjustments for 11–17 year olds; ^Δ^ missing data due to missing height or weight information (*n* = 816, weighted).

**Table 3:** Reactions to the fast-food advert and associations with demography and the BMI group.

Reactions to the Fast-Food (McDonald’s) Advert																	
		Seemed Popular		Age Appeal		Product Fun		Advert Fun		Product Appealing		Liked Advert		Product Healthy		Temptation to Try	
Variables	*n*	*AOR*	*p*	*AOR*	*p*	*AOR*	*p*	*AOR*	*p*	*AOR*	*p*	*AOR*	*p*	*AOR*	*p*	*AOR*	*p*
**Age**																	
16–19 years old	1323	Ref	-	Ref	-	Ref	-	Ref	-	Ref	-	Ref	-	Ref	-	Ref	-
11–15 years old	1064	0.64	<0.001	1.33	0.001	0.97	0.70	0.95	0.54	0.88	0.14	1.33	0.002	0.94	0.51	1.53	<0.001
**Gender**																	
Male	1139	Ref	-	Ref	-	Ref	-	Ref	-	Ref	-	Ref	-	Ref	-	Ref	-
Female	1248	1.46	<0.001	1.35	<0.001	1.60	<0.001	1.83	<0.001	1.43	<0.001	1.52	<0.001	1.01	0.94	1.25	0.01
**Ethnicity**																	
Other	398	Ref	-	Ref	-	Ref	-	Ref	-	Ref	-	Ref	-	Ref	-	Ref	-
White British	1989	1.19	0.17	1.16	0.20	1.00	1.00	0.96	0.69	0.97	0.77	0.91	0.42	0.96	0.72	1.08	0.53
**Country**			0.88		0.42		0.76		0.44		0.57		0.81		0.10		0.40
England	1809	Ref	-	Ref	-	Ref	-	Ref	-	Ref	-	Ref	-	Ref	-	Ref	-
Wales (*vs. England*)	184	1.09	0.63	1.28	0.12	1.19	0.29	1.08	0.64	1.02	0.90	1.01	0.96	0.91	0.62	1.10	0.59
Scotland (*vs. England*)	275	0.92	0.56	0.95	0.68	0.99	0.94	1.13	0.35	1.13	0.36	1.03	0.81	1.36	0.03	1.20	0.18
N. Ireland (*vs. England*)	119	1.04	0.85	1.09	0.67	1.02	0.93	1.32	0.16	1.25	0.25	1.21	0.33	1.25	0.28	1.26	0.25
**IMD**			0.30		0.29		0.32		0.40		0.74		0.34		0.07		<0.001
1	397	Ref	-	Ref	-	Ref	-	Ref	-	Ref	-	Ref	-	Ref	-	Ref	-
2 (*vs. 1*)	490	0.91	0.54	1.02	0.89	0.98	0.91	1.04	0.78	0.91	0.48	0.97	0.84	1.14	0.38	0.85	0.27
3 (*vs. 1, 2*)	515	1.30	0.04	1.12	0.33	0.97	0.80	1.05	0.68	0.96	0.70	1.11	0.37	0.84	0.15	0.79	0.05
4 (*vs. 1, 2, 3*)	570	0.99	0.96	0.82	0.05	0.81	0.04	0.89	0.27	0.89	0.24	0.81	0.06	0.89	0.32	0.64	<0.001
5 (*vs. 1, 2, 3, 4*)	415	0.96	0.75	1.03	0.81	1.05	0.67	1.19	0.12	1.02	0.84	1.00	0.98	0.77	0.03	0.94	0.60
**Weight Status**			0.02		0.30		0.03		0.27		0.10		0.08		0.17		0.15
Underweight	416	Ref	-	Ref	-	Ref	-	Ref	-	Ref	-	Ref	-	Ref	-	Ref	-
Healthy weight (*vs. u/w^4^*)	1503	1.28	0.04	1.16	0.19	1.15	0.21	1.11	0.34	1.16	0.18	1.10	0.44	1.26	0.07	1.05	0.71
Overweight (*vs. u/w & h’lthy*)	351	1.25	0.11	1.19	0.15	1.33	0.02	1.11	0.41	1.29	0.04	1.19	0.17	0.96	0.79	1.07	0.63
Obese (*vs. all other*)	117	1.70	0.04	1.18	0.41	1.40	0.10	1.39	0.10	1.25	0.26	1.53	0.03	1.32	0.18	1.56	0.03

Notes: Dependent variable for all models: did the participant have a positive reaction (codes 4/5) or a neutral and negative reaction (codes 1–3); Hosmer and Lemeshow for all models *p* > 0.05; *AOR* = Adjusted Odds Ratio; cases with missing data on one or more variables in all models (*n* = 961, i.e., could not watch video or report BMI).

**Table 5:** Reactions to the confectionery advert and associations with demography and the BMI group.

		Reactions to the Confectionery (Haribo) Advert															
		Seemed Popular		Age Appeal		Product Fun		Advert Fun		Product Appealing		Liked Advert		Product Healthy		Temptation to Try	
Variables	*n*	*AOR*	*p*	*AOR*	*p*	*AOR*	*p*	*AOR*	*p*	*AOR*	*p*	*AOR*	*p*	*AOR*	*p*	*AOR*	*p*
**Age**																	
16–19 years old	1341	Ref	-	Ref	-	Ref	-	Ref	-	Ref	-	Ref	-	Ref	-	Ref	-
11–15 years old	1076	0.74	0.001	1.79	<0.001	0.98	0.81	0.93	0.40	1.09	0.29	1.20	0.03	2.22	<0.001	1.57	<0.001
**Gender**																	
Male	1146	Ref	-	Ref	-	Ref	-	Ref	-	Ref	-	Ref	-	Ref	-	Ref	-
Female	1271	1.37	<0.001	1.19	0.04	1.34	<0.001	1.22	0.03	1.40	<0.001	1.40	<0.001	0.92	0.55	1.27	0.005
**Ethnicity**																	
Other	405	Ref	-	Ref	-	Ref	-	Ref	-	Ref	-	Ref	-	Ref	-	Ref	-
White British	2012	1.07	0.58	1.11	0.36	1.00	0.98	1.05	0.67	1.13	0.28	1.31	0.02	0.71	0.05	0.97	0.82
**Country**			0.13		0.18		0.43		0.07		0.15		0.01		0.06		0.16
England	1830	Ref	-	Ref	-	Ref	-	Ref	-	Ref	-	Ref	-	Ref	-	Ref	-
Wales (*vs. England*)	186	1.23	0.26	1.17	0.32	1.05	0.77	1.12	0.51	1.19	0.27	0.91	0.55	1.21	0.45	1.29	0.10
Scotland (*vs. England*)	281	0.79	0.10	0.79	0.07	0.82	0.13	0.72	0.01	0.78	0.06	0.65	0.001	0.87	0.55	0.95	0.72
N. Ireland (*vs. England*)	120	1.24	0.33	0.95	0.79	1.06	0.75	0.92	0.69	1.03	0.87	0.99	0.97	1.88	0.01	1.35	0.12
**IMD**			0.55		0.43		0.08		0.39		0.72		0.40		0.16		0.19
1	405	Ref	-	Ref	-	Ref	-	Ref	-	Ref	-	Ref	-	Ref	-	Ref	-
2 (*vs. 1*)	495	0.83	0.20	0.86	0.25	0.80	0.10	0.93	0.59	0.86	0.27	0.80	0.11	0.96	0.83	0.97	0.80
3 (*vs. 1, 2*)	523	1.12	0.36	1.04	0.72	1.30	0.02	1.24	0.08	0.99	0.92	1.11	0.37	0.74	0.10	0.93	0.49
4 (*vs. 1, 2, 3*)	571	1.01	0.92	0.85	0.12	1.01	0.96	0.94	0.56	0.94	0.56	0.95	0.58	0.71	0.06	1.04	0.68
5 (*vs. 1, 2, 3, 4*)	423	1.07	0.60	0.99	0.90	0.97	0.78	1.05	0.70	0.91	0.40	1.04	0.73	0.99	0.96	0.77	0.02
**Weight Status**			0.09		0.02		0.11		0.16		0.03		0.12		0.40		0.81
Underweight	415	Ref	-	Ref	-	Ref	-	Ref	-	Ref	-	Ref	-	Ref	-	Ref	-
Healthy (*vs. u/w^4^*)	1524	1.16	0.23	0.99	0.96	1.02	0.86	1.05	0.66	1.14	0.24	0.91	0.43	1.05	0.78	1.04	0.71
Overweight (*vs. u/w & h’lthy*)	359	1.04	0.76	1.12	0.34	1.18	0.19	1.04	0.78	1.37	0.01	1.14	0.28	1.22	0.30	0.95	0.71
Obese (*vs. all other*)	119	1.78	0.02	1.72	0.01	1.51	0.06	1.67	0.03	1.32	0.17	1.38	0.11	1.46	0.19	1.18	0.40

Notes: Dependent variable for all models: did the participant have a positive reaction (codes 4/5) or a neutral and negative reaction (codes 1–3); Hosmer and Lemeshow for all models *p* > 0.05; *AOR* = Adjusted Odds Ratio; cases with missing data on one or more variables in all models (*n* = 931, i.e., could not watch video or report BMI).
